# The PET@home Toolkit: A Process Evaluation Study

**DOI:** 10.3390/ani14233475

**Published:** 2024-12-02

**Authors:** Peter W. A. Reniers, Karin Hediger, Ine J. N. Declercq, Marie-José Enders-Slegers, Debby L. Gerritsen, Ruslan Leontjevas

**Affiliations:** 1 Faculty of Psychology, Open Universiteit, 6149 AT Heerlen, The Netherlands; peter.reniers@ou.nl (P.W.A.R.); karin.hediger@ou.nl (K.H.); ine.declercq@ou.nl (I.J.N.D.); marie-jose.enders@ou.nl (M.-J.E.-S.); 2 Faculty of Behavioural Sciences and Psychology, University of Lucerne, 6002 Luzern, Switzerland; 3 Department of Primary and Community Care, Radboud Alzheimer Centre, Radboud Institute for Health Sciences, Radboud University Medical Center, 6500 HB Nijmegen, The Netherlands; debby.gerritsen@radboudumc.nl; 4 Frailty in Ageing (FRIA) and Mental Health and Wellbeing (MENT) Research Group, Department of Gerontology, Faculty of Medicine and Pharmacy, Vrije Universiteit Brussel, 1050 Brussels, Belgium

**Keywords:** process-evaluation, PET@home toolkit, companion-animals, long-term care, home care, older adults

## Abstract

The Dutch PET@home Toolkit was developed to mitigate challenges, foster conversations, and support planning concerning pets in long-term care at home. This process evaluation study aimed to evaluate the practical application of the toolkit on four topics: (1) satisfaction; (2) relevance; (3) feasibility; and (4) integration of the PET@home Toolkit materials in home care. Outcomes may be used to improve the toolkit materials to better align with the long-term care at home setting. Accounting for data saturation, professional caregivers (N = 6), clients (N = 2), and family caregivers (N = 2) who used toolkit materials participated in semi-structured interviews. Interviews were analyzed by two researchers in ATLAS.ti using an inductive-iterative approach. The researchers reached a consensus on themes and clustering within interview topics. This led to the identification of the following themes pertaining to each interview topic: satisfaction (general impression, suggestions for improvement); relevance (awareness, planning, pet-related aspects in practice, impact on healthcare quality); feasibility (healthcare practice, competence, quantity); and implementation (digitalization, task owner, piloting, timing). Several improvements were made to toolkit materials, such as providing clearer instructions for clients in the information booklet. Participants acknowledged the toolkit could lead to better planning while mitigating potential challenges concerning pets, which may lead to longer-lasting relationships between clients and their pets.

## Simple Summary

In the Netherlands, we created the PET@home Toolkit to help people receiving long-term care at home, their families, and professional caregivers to talk about and plan for their pets. It aims to support the beneficial bond people who are receiving long-term care at home experience with their pets. This study looked at the use of the toolkit, focusing on four topics: (1) satisfaction; (2) relevance; (3) feasibility; and (4) integration of the PET@home Toolkit in home care. We interviewed professional caregivers (N = 6), people receiving home care (N = 2), and family caregivers (N = 2) who used the materials. Two researchers analyzed the interviews and identified the following themes pertaining to each topic: satisfaction (general impression, suggestions for improvement); relevance (awareness, planning, pet-related aspects in practice, impact on healthcare quality); feasibility (healthcare practice, competence, quantity); and integration (digitalization, task owner, piloting, timing). As a result, some changes were made to the materials, such as adding clearer instructions in the information booklet for people receiving long-term care at home. Participants indicated that the toolkit could lead to better planning and solve some challenges concerning pets. This could potentially lead to longer-lasting relationships between people receiving home care and their pets, benefitting the well-being of both.

## 1. Introduction

Clients receiving long-term care at home, a group that mainly consists of older adults, often indicate that their pets play an important role in their well-being [1,2,3]. Therefore, it is essential to support the beneficial relationships that clients experience with their pets. To assist clients with pets, as well as family caregivers and professional caregivers in maintaining these relationships, we developed the PET@home Toolkit in the Netherlands (the Dutch toolkit is publicly available via www.ukonnetwerk.nl/tools/pet-home accessed on 27 November 2024) using a participatory research approach [4]. The toolkit includes an information booklet, leaflets on animal welfare and communication, an infographic, and a checklist for care plan discussions—designed to enhance understanding, communication, awareness of challenges, and planning for pet care in long-term care at home settings [4].

This article describes a process evaluation that was conducted after a small group of professional caregivers used the PET@home Toolkit in practice. Process evaluations, often using qualitative methods, are valuable for understanding how new healthcare-supportive materials work in context and for identifying effective implementation strategies [5,6,7]. While clients, family caregivers, and professional caregivers contributed to the toolkit’s development, a process evaluation after practical use can further help to refine the materials, ensuring they are well suited to the complexities of home care.

The toolkit’s development followed the Medical Research Council (MRC) framework for developing and evaluating complex interventions [8]. This framework emphasizes the importance of stakeholder involvement and an iterative process comprising four stages: (1) developing the intervention; (2) assessing its feasibility; (3) evaluating the intervention; and (4) implementing the intervention [8]. Our previous publication describes the first step within the MRC framework [4], while this study focuses on the feasibility and evaluation phase (phases 2 and 3), informed by practical application and feedback from users.

A process evaluation can provide additional insights into the complexities of home care. These complexities can arise from the involvement of various stakeholders, including clients with pets, family caregivers, and professional caregivers. Each group possesses unique characteristics, such as personality, education level, financial resources, location, social support networks, and healthcare needs [9,10,11,12]. The pet’s characteristics, such as its species (e.g., dog or cat) or physical and behavioral issues [13,14], can add to the caregiving burden, primarily affecting family caregivers [15]. These complexities necessitate tailored support, such as assistance with dog walking or cleaning a cat’s litterbox and are further complicated by the varying availability of local support options (e.g., volunteers). Therefore, involving clients and family caregivers in healthcare decision-making using a person-centered care approach while considering animal well-being is crucial [16]. The PET@home Toolkit can support stakeholders in long-term care at home in this endeavor.

This study aimed to evaluate the practical application of the PET@home Toolkit by assessing stakeholder feedback on four key aspects: (1) satisfaction; (2) relevance; (3) feasibility; and (4) integration of the PET@home Toolkit materials in long-term care at home. By focusing on these areas, the evaluation may provide insights for further improving the toolkit to better address the complex needs of stakeholders in long-term care at home.

## 2. Materials and Methods

### 2.1. Study Design

This study was based on a process evaluation framework for long-term care settings [17]. The primary focus was on the quality of the PET@home Toolkit by assessing the feasibility, relevance, and satisfaction of the materials, as well as strategies to foster the integration of the toolkit materials into long-term care at home.

### 2.2. Researcher Characteristics

The research group consisted of two PhD students—one specializing in human-animal bond research (PR) and one in geriatric care research (ID)—and a psychology master’s student intern (JK) who also works at a home care organization. The research process was guided by two expert supervisors in human-animal bond research (KH and ME) and two in geriatric care research (RL and DG). The research group held regular discussions, providing sufficient opportunities for reflection.

### 2.3. Participants and Procedures

Participants were recruited between August 2023 and January 2024 through three community care organizations active in long-term care at home, which distributed our information letter to their staff. Professional caregivers working in home care (e.g., case managers and nurses), clients receiving long-term care at home, and their family caregivers, all of whom had used toolkit materials, were invited to participate in individual semi-structured interviews. Initially, eight interviews were conducted, followed by two additional interviews to confirm the data saturation [18].

After expressing interest in participating, professional caregivers received a brief online or in-person introduction to the toolkit materials. Subsequently, these professional caregivers invited clients with pets, whom staff considered sufficiently cognitively competent, and family caregivers, through purposive sampling [19], to test the PET@home Toolkit materials and provide written consent to be contacted by a researcher. Interviews were conducted through Microsoft Teams (N = 7), telephone (N = 2), or in-person (N = 1) and ranged from 19 to 59 min in duration. JK conducted eight interviews, and PR conducted 2. Participants received a 20-euro gift voucher (e.g., for flowers) as an incentive.

### 2.4. Process Evaluation Interview Protocol

As followed from the applied process evaluation framework, the interview protocol covered satisfaction (e.g., ‘What was your general impression of the toolkit?’), relevance (e.g., ‘What elements of the toolkit were less relevant according to you and why?’), feasibility (e.g., ‘What challenges did you encounter using the toolkit?’), and implementation (e.g., ‘How do you think the toolkit can best be integrated into healthcare?’). Participants were also asked to rate satisfaction, relevance, and feasibility from 1 (very poor) to 10 (excellent) (e.g., ‘How would you rate the usability of the toolkit materials in practice?’). See Appendix A for the full interview protocol. Demographic information, such as gender, age, and type of pet, was collected to describe participants.

### 2.5. Data Analysis

Transcripts were analyzed using open coding and an iterative-inductive approach by PR and JK with ATLAS.ti for Windows. Initially, the two researchers independently analyzed eight transcripts and then discussed codes until reaching a consensus on the themes. Two additional interviews, one with a family caregiver and one with a professional caregiver, were conducted to determine data saturation. Subsequently, the two researchers independently clustered the themes deductively within the four interview topics, followed by a second discussion to reach a consensus on the clustering.

### 2.6. Ethical Considerations

The study protocol was approved by the research ethics committee of the Open Universiteit of The Netherlands (U202206075). All participants provided written informed consent. Audio and video recordings were deleted once the project was completed, and transcripts were pseudonymized and stored securely, separate from personal information, on the Open Universiteit drive.

## 3. Results

### 3.1. Participant Characteristics

Professional caregivers (N = 6), clients receiving long-term care at home (N = 2), and family caregivers (N = 2) participated in this study. Among the professional caregivers, four were case managers, two were nurses, five were female, five had completed higher education, and one held a university degree. Both participating clients were male and had lived with a dog for approximately nine years. The family caregivers included one male and one female; one of their care recipients lived with a dog, while the other lived with a cat. Additional participant details are provided in Table 1. An overview of the themes identified within the interview topics is presented in Table 2.

### 3.2. Satisfaction

Satisfaction ratings ranged from 4 (N = 1) to 9.5 (N = 1), with a mode of 8 (N = 5). The person who gave the lowest rating indicated that the toolkit contained too much information and too many materials. Two themes were identified within this topic: *general impression* and *suggestions for improvement*.

#### General Impression and Suggestions for Improvement

Most participants responded positively to the toolkit materials. They appreciated the design, images, and clear language that was easy to understand. The large font size and A4 format of the information booklet (A4) were appreciated, making it easy to read for individuals with poor eyesight.

CL: *‘What is written is all fine. It’s all very clear, easy to read, and it’s even a nice little book as well.’*

Participants provided a few suggestions for improvement. For instance, a professional caregiver expressed discomfort with the negative phrasing of the statements in the care plan discussions checklist (e.g., ‘The client has physical disabilities that can impact pet care).

PC: *‘Only that it was occasionally uncomfortable [for me] because those questions were formulated negatively, umm… negative might not be the right word.’*

### 3.3. Relevance

The ratings of the relevance ranged from 6 (N = 1) to 9 (N = 2), with the mode being 7 (N = 3). Four themes were identified within this topic: *awareness*, *mutual understanding*, *pets in healthcare practice*, and *healthcare quality*.

#### 3.3.1. Awareness

Participants noted that the toolkit materials raised awareness about pets and the associated challenges. Even when not using the materials, they more often discussed pets with their clients and colleagues.

PC: *‘Well, I think it’s about raising awareness… so also with the client, how do you care for [the pet]. Of course, we also sometimes see neglect [of pets] … For us, it’s also a reminder of… Well, that we should be able to discuss this too… and do something about it.’*

#### 3.3.2. Planning

Conversations and planning, such as using the poster in the information booklet to record and share agreements about pets, fostered mutual understanding and provided reassurance to participants, reducing worries about potential misunderstandings. For example, a pet may need to be rehomed, and a client might assume that one of their children or neighbors will take on this responsibility, even though it has never been discussed.

PC: *‘If you fill out one of those posters to record agreements with [in the information booklet], it might be clear for everyone that this has been discussed … For instance, if the pet becomes sick… who will take it to the vet? Or if someone [the client] must go to the hospital, then who will [care for the pet]? She said… Yes, the neighbor has done it before, and she did, and I think she will do it again… But you never really know if she will come back from the hospital, and then you have a problem.’*

#### 3.3.3. Pet-Related Aspects in Practice

Several examples of challenges concerning pets in healthcare practice were provided. Additionally, professionals, clients, and family members acknowledged the importance of pets for clients and their mental, social, and physical benefits and emphasized the need to pay attention to clients’ pets. A client expressed that she would not allow a professional caregiver who was not fond of pets into her home. A professional caregiver indicated rehoming one of her client’s pets was very time-consuming, and although it was not part of her job description, she felt responsible for the well-being of both the client and the pet. These examples highlight the importance of accounting for the presence of pets in practice.

FC: *‘We got a case manager. He stood at the door and remained at the door. He didn’t come in because we had two dogs, and he didn’t want that. He wanted us to lock them up. I’m not going to do that. I said to him: You can come in, or you can leave. Then he left. That’s an example where I think that’s not right.’*

#### 3.3.4. Impact on Healthcare Quality

Most of the participants indicated that using the toolkit had a positive impact on healthcare quality, particularly in building relationships between professional caregivers and clients with pets. This suggests that discussing the presence of pets with clients and their relatives is relevant. One professional caregiver noted that discussing a pet with a client provided additional insight into the client’s functioning, providing an opportunity to improve healthcare quality. For instance, the client usually would not let caregivers check her refrigerator, but when asked about her cat’s food, she opened it, allowing professional caregivers to check the contents (e.g., for expired food). The checklist for care plan discussions intended to be used by professional caregivers [4] may be useful in this context.

PC: *‘It also struck me how, how little [the client] could actually tell about [the care of the pet], well, just in general when I asked questions about [the pet]. That really struck me, I thought oh, then it’s not going as well [with the client’s health] as I… Well, as I previously thought.’*

### 3.4. Feasibility

Eight participants rated the usability of the toolkit in practice, while two participants did not provide a rating: one family caregiver was not asked to rate practicality, and one professional caregiver began elaborating on her client’s cognitive decline, as later discovered in the transcripts. Ratings varied between 4 (N = 1) and 9 (N = 1), with the modus being 8 (N = 4). The low rating of 4 provided by a professional caregiver was attributed to the amount of information in the toolkit, which was considered excessive. Three themes emerged within this topic: *competence*, *quantity*, and *healthcare practice*.

#### 3.4.1. Competence

Participants generally felt competent to use the materials. However, one family caregiver expressed confusion due to a lack of guidance from the professional caregiver who provided the information booklet.

FC: *‘It is not confusing. It is a beautiful little book, children can read it, but I was not sure what to do with it. That was the point.’*

#### 3.4.2. Quantity

Some participants felt overwhelmed by the number of materials and expressed concern that using everything with every client with pets would be time-consuming. However, most participants selected only the relevant materials for each client.

PC: *‘I found it a lot. It was quite a stack. I’ve sorted out what’s for the client … and what’s for me. That was already a task. And I found it a lot.’*

#### 3.4.3. Healthcare Practice

Some participants mentioned that understaffing and time management issues in daily healthcare practice could hinder the use of toolkit materials. One participant indicated that their day mainly consisted of managing daily issues and crises, making the use of the toolkit a lower priority for many professional caregivers. Another important issue raised was the difficulty of communicating with clients living with dementia.

FC: *‘Well, things often disappear with a person living with dementia, and none of the children have seen [the information booklet] lying around, so I think, I fear it might have ended up with the wastepaper.’*

### 3.5. Integration in Long-Term Care at Home Practice

Professional caregivers shared their insights on integrating the toolkit in the long-term care at home practice, revealing four themes: *piloting*, *timing of application*, *digitalization*, and *responsibility*. The outcomes of this interview topic were used to update the implementation guide that accompanies the toolkit.

#### 3.5.1. Piloting

Participants confirmed that new materials are often piloted with a small team of enthusiastic caregivers before being scaled up within the organization.

PC: *‘Yes, usually this is set up by policy or by project leaders who then come up with a plan and first discuss it with us. Then, of course, you start with a few areas where the pilot takes place, and then it gets rolled out further.’*

#### 3.5.2. Timing of Application

Professional caregivers emphasized the importance of using the toolkit early on with new clients with pets. Ideally, it should be introduced during the initial intake or shortly thereafter to help mitigate potential challenges.

PC: *‘So maybe I wouldn’t bring it up during the first conversation, but somewhere early on. Like, hey, we’ve had our first conversation, we’ve set some actions in motion, and now that things have calmed down a bit, I see, I’ve noticed before, but [pet’s name] is also around. Should we talk about that as well?’*

#### 3.5.3. Task Owner

Participants noted that case managers and home care workers may not always be the best suited to use the toolkit materials. They suggested that support workers, who are often involved earlier and look after the individual needs of clients, may be better equipped for this role.

PC: *‘We also work with support workers, so we have a dedicated support team, and they sometimes get involved earlier than we are … if it’s really about things like how to organize your life, how to deal with family or finances, we are not the first to be called in. I think, for example, the support team often quickly encounters a pet.’*

#### 3.5.4. Digitalization

Participants suggested integrating relevant information concerning pets into the digital care planning systems to make it more accessible. They recommended using the care plan discussions checklist as a foundation for registering information about pets in the digital care planning system.

PC: *‘We are incredibly digital, so I think digitalization is very important. We are moving away from paper. I think the paper booklet is very nice, but we should just have it on hand to take with us whenever we see that clients have pets.’*

## 4. Discussion

### 4.1. General Discussion

The aim of this process evaluation was to gather feedback from stakeholders on the quality of the toolkit materials, focusing on (1) satisfaction; (2) relevance; (3) feasibility; and (4) strategies to integrate the toolkit materials into the long-term care at home practice. These insights have been used to adapt the toolkit materials to better align with daily home care practices.

Overall, participants expressed satisfaction with the toolkit and its materials, though some also suggested some improvements. One participant indicated feeling uncomfortable with negative statements in the PET@home care plan discussion checklist. Positive or negative statements may lead to framing effects, potentially influencing clients’ responses [20]. Therefore, we considered it important for the care plan discussion checklist to contain neutral questions. The checklist’s statements were consequently reformulated. For instance, we changed the statement ‘The client has physical disabilities that can impact pet care, such as problems with walking, feeding, cleaning or caring for the pet’ to ‘Is the client sufficiently physically capable of independently caring for the pet?) where applicable. Additionally, the challenge of communicating with clients who have dementia suggests that, ideally, a family caregiver should be present during discussions about the pet’s care. The care plan discussion checklist and the information booklet can be used to record agreements between stakeholders.

Participants noted that the toolkit raised awareness about the benefits and challenges related to pets, which could be a starting point for improving the quality of the healthcare provided to clients with pets. An important aspect of this is the communication between stakeholders about pets, using a person-centered care approach while considering animal well-being [16]. This supports the relevance of the toolkit to long-term care at home. Since many people consider their pets as family members [21], most clients will likely appreciate having their pets considered in their care. Agreements and planning concerning pets can bring clarity to clients, their families, and professional caregivers, potentially fostering mutual understanding, improving healthcare quality, and reducing the burden on those involved. Therefore, healthcare organizations should consider documenting pet-related agreements in a digital care plan. This can enhance communication, decision-making, and accountability among stakeholders [22]. Moreover, using the toolkit for clients with pets aligns well with the person-centered care paradigm, which is considered a benchmark for healthcare quality [23]. Person-centered care tailors caregiving to clients’ needs and wishes, actively involving them in their care [16,24]. The results suggest that it is important for clients to receive guidance from a professional caregiver when using the information booklet. However, professional caregivers mentioned time constraints as a barrier to utilizing the toolkit materials. Time pressure is a common issue in home care [25,26,27], which may affect the amount of guidance a professional caregiver can provide when offering a client the information booklet. To address this, we added additional instructions in the booklet’s introduction and table of contents. These changes may offer clients and family caregivers extra support in using the materials independently. Additionally, some professional caregivers indicated that the toolkit included a lot of materials. However, some of the materials, such as the communication and animal welfare leaflets, are designed for professional caregivers rather than clients. Therefore, not all materials are required to be used with clients. We trust that caregivers can assess and select the materials that are necessary for each specific situation.

Time pressure may also hinder integrating the toolkit in home care settings. While using the toolkit does not necessarily require much time. Its use can start with providing clients with pets and their relatives with the information booklet and explaining its purpose. Nevertheless, professional caregivers, under time constraints, may be reluctant to adopt innovations. According to the ‘Diffusion of Innovations’ theory [28,29], successful implementation often relies on early adopters—those enthusiastic about the anticipated benefits [28,30]. To encourage wider use of an intervention or new materials, it is essential to showcase positive user experiences, such as the time required to use the toolkit [28,30]. Therefore, piloting the PET@home Toolkit with enthusiastic early adopters could promote its adoption across home care organizations before wider and more sustainable adoption in long-term care at home [31]. To support the integration of the toolkit in home care services, we developed an implementation guide to facilitate its use.

### 4.2. Limitations, Strengths, and Future Research

We identified a few limitations in this study. The first limitation was that only ten interviews were conducted, with limited participation from clients (N = 2) and family members (N = 2). However, we applied data saturation principles during the analysis by first creating a set of themes based on eight interviews and subsequently conducting and analyzing two additional interviews to determine saturation. Additionally, the involvement of clients, family caregivers, and professional caregivers was crucial throughout the entire project, leading to the development of the toolkit and placing the toolkit on a foundation of both practical experience and scientific research [1,4].

A second limitation was that the toolkit materials might not be generalizable to long-term care at home settings in other countries, as they were developed specifically for the Dutch context. Different countries have varying healthcare systems and challenges related to living with pets. Therefore, the materials would need to be adapted accordingly.

Nevertheless, this study also has notable strengths. We successfully used a process evaluation framework designed for randomized clinical trials (RCTs) of clinical interventions in long-term care settings [17]. Since we did not conduct an RCT or create a clinical intervention, in this study, we used a part of the model that focused on the quality (specifically on satisfaction, relevance, and feasibility) and strategies for integrating the PET@home Toolkit into practice, various relevant suggestions were provided and used to improve the materials, ensuring better alignment with daily long-term home care practices.

Future research is needed to evaluate the effectiveness of the toolkit in home care practice, particularly its impact on client health outcomes, such as perceived quality of life and animal well-being. It would also be useful to compare the experiences of clients in organizations that implement the toolkit with those that do not. Furthermore, interviewing professional caregivers about their experiences with using the toolkit over a prolonged period could offer additional insights, such as the time investment required for using the toolkit, which may vary depending on each individual client.

The current interest from organizations outside the Netherlands suggests the potential for adapting the PET@home Toolkit for international use. Research, such as focus groups with clients, family caregivers, professional caregivers, and animal welfare experts in other countries, would be needed to identify any necessary adaptations for use abroad.

## 5. Conclusions

Overall, participants expressed positive feedback about the PET@home Toolkit and its materials while also providing various insights that led to improving the toolkit to better align with long-term care at home settings. Additionally, our study demonstrated that a process evaluation framework initially designed for RCTs could be adapted to assess the practical application of a non-clinical intervention in long-term care at home settings.

The role of pets experienced by clients receiving long-term care at home and their potential to improve healthcare quality underscore the importance of considering clients’ pets with the PET@home Toolkit. The Dutch versions of the materials are publicly available through the University Knowledge Network for Older Adult Care Nijmegen (www.ukonnetwerk.nl accessed on 27 November 2024) and the Open Science Framework (https://doi.org/mxh2 accessed on 27 November 2024).

## Figures and Tables

**Table 1:** Participant Characteristics.

Professional Caregivers	Age Range	Gender	Function	Level of Education
PC1	26–30	female	case manager	higher education
PC2	26–30	female	case manager	higher education
PC3	51–55	female	case manager	higher education
PC4	51–55	male	case manager	higher education
PC5	26–30	female	nurse	higher education
PC6	26–30	female	nurse	university
**Clients and Family Caregivers**	**Age Range**	**Gender**	**Type of Pet (Age in Years)**	**Level of Education**
CL1	76–80	male	dog (9)	elementary school
CL2	81–85	male	dog (9.5)	higher education
FC1	51–55	male	cat (N/A)	university
FC2	56–60	female	dog (7)	secondary education

N/A—not applicable.

**Table 2:** Topics and Themes.

Satisfaction	Relevance
general impression suggestions for improvement	awareness planning pet-related aspects in practice impact on healthcare quality
**Feasibility**	**Integration in Practice**
competence quantity healthcare practice	piloting timing task owner digitalization

## Data Availability

Data is available on request at peter.reniers@ou.nl.

## Appendix A

### Interview Protocol

Satisfaction

- What is your overall impression of the toolkit?
- On a scale of 1–10, how satisfied were you?
- In what ways could we improve this score?

Relevance

- What is your experience regarding the relevance of the toolkit?
- How would you rate the relevance of the toolkit from 1 to 10?
- In what ways has the toolkit contributed to the quality of (received) care?
- In what ways has the toolkit helped the relationship between caregiver–client, informal caregiver–client, or pet–client?
- Which materials of the toolkit have left the most lasting impression on you? Can you explain why these elements stood out?
- Which materials of the toolkit do you find less relevant? What makes these materials less relevant to you?

Feasibility

- What is your experience regarding the use of the toolkit?
- How would you rate the usability of the toolkit materials in practice from 1 to 10?
- What knowledge and experiences did you have on this topic beforehand?
- In what ways has the toolkit supported you?
- What were your expectations regarding the use of the toolkit? Were these expectations met?
- What problems did you encounter while using the toolkit?
- For care staff: Did you feel sufficiently capable of using the toolkit? Can you elaborate?
- Is there a difference between what was “planned on paper” and what you carried out? (Did you do things differently?)

Implementation

- How do you see the toolkit fitting within your current role (for care staff) or in your life (for clients)?
- How do you think the toolkit can best be integrated into care?
